# Validity of the Health Belief Model Applied to Influenza among people with chronic diseases: Is it time to develop a new knowledge domain?

**DOI:** 10.1371/journal.pone.0274739

**Published:** 2022-09-15

**Authors:** Sladjana Arsenović, Goran Trajković, Tatjana Pekmezović, Tatjana Gazibara

**Affiliations:** 1 Department of Immunization, Public Health Institute of Republic of Srpska, Regional Center Foča, Foča, Republic of Srpska (Bosnia and Herzegovina); 2 Institute of Medical Statistics and Informatics, Faculty of Medicine, University of Belgrade, Belgrade, Serbia; 3 Institute of Epidemiology, Faculty of Medicine, University of Belgrade, Belgrade, Serbia; Bay Area Hospital, North Bend Medical Center, UNITED STATES

## Abstract

There is a lack of comprehensive instruments for the assessment of compliance with influenza immunization. The purpose of this study was to examine psychometric characteristics of the Health Belief Model Applied to Influenza (HBMAI) among people with chronic diseases. We selected people residing in four municipalities of the Foča region (Republic of Srpska—Bosnia and Herzegovina) who were listed in the official records to receive the recommended influenza immunization in 2017/2018. Participants were interviewed using the HBMAI questionnaire at their homes. The HBMAI is composed of 45 items classified in 7 domains (Susceptibility, Seriousness, Benefits, Barriers, Knowledge, Health Motivation and Cue to Action). The confirmatory factor analysis (CFA) suggested that the Serbian HBMAI did not fit the original structure. The parallel analysis suggested that HBMAI in Serbian had 6 domains, instead of the original 7. The domain of "Knowledge" was removed. The domains of "Barriers", "Health Motivation" and "Cue to Action" preserved their original structure. The domains of "Susceptibility", "Seriousness" and "Benefits" were partially modified. The parameters on the CFA for the new modified HBMAI in Serbian were acceptable (goodness of fit index [GFI] = 0.946, comparative fit index [CFI] = 0.967, Tucker-Lewis index [TLI] = 0.963, root mean square error of approximation [RMSEA] = 0.044 and standardized root mean square residual [SRMR] = 0.078). This modified HBMAI version with 6 domains, not including the Knowledge domain, is recommended for use in research about influenza among people with chronic diseases in Serbian language.

## Introduction

Influenza may be a serious infection among people who have chronic illnesses, because the underlying health status makes them prone to health complications, more severe clinical course and poor outcomes [1]. Immunization against influenza is well-acknowledged to lessen the risk of hospitalizations, complications and related mortality [2]. For this reason, people who have chronic illnesses are identified as a priority risk group for influenza immunization [3].

In Republic of Srpska (Bosnia and Herzegovina) in 2018, influenza was one of the most commonly diagnosed infections [4]. Immunization against influenza is recommended to people with chronic diseases and it is free of charge for these individuals [4]. Based on the review of records of the Public Health Institute of Republic of Srpska (Bosnia and Herzegovina), the influenza immunization coverage is approximately 80% (unpublished data). This means that there are still people who are at high risk of developing severe forms of influenza and who fail to comply with vaccination. To gain a more thorough insight into the background of non-compliance to influenza immunization, it would be helpful to conduct a survey about beliefs, motivations and barriers behind the decision to skip the recommended immunization [5–7]. However, there is a lack of valid and comprehensive questionnaires.

The Health Belief Model Applied to Influenza is a questionnaire which examines various dimensions behind behaviors related to immunization against influenza. It has been previously used in adults population [8] and among health care workers [9, 10]. The psychometric testing suggested that this questionnaire is a valid instrument to test beliefs about influenza. The purpose of this study was to examine psychometric characteristics of the Health Belief Model Applied to Influenza in a population of people with chronic diseases.

## Materials and methods

### Setting and participants

After disintegration of former Yugoslavia due to the armed conflict (1991–1995), the territory of Bosnia and Herzegovina was divided in two entities and one district based on the Agreement for Peace in Bosnia and Herzegovina [11]. One entity is the Federation of Bosnia and Herzegovina (Federation) and the other is Republic of Srpska. In Republic of Srpska, the majority of the population is Serbian. The two entities differ in planning and delivery of health care (i.e. both entities have their own Ministry of Health). The Public Health Institute of Republic of Srpska is organized through five regional centers located in towns of Doboj, Trebinje, East Sarajevo, Zvornik and Foča.

The Public Health Institute in the region of Foča is in charge of public health activities in six local municipalities (towns of Foča, Novo Goražde, Višegrad, Rudo, Čajniče and Kalinovik). We selected community-dwelling people residing in four municipalities of the Foča region (Foča, Novo Goražde, Višegrad and Rudo), because the records showed that non-vaccinted people in season 2017/2018 lived in these four towns. The inclusion criteria were: being diagnosed with chronic illness, being scheduled for influenza vaccination according to the official records of the Institute and signed written consent for participation. The exclusion criteria were: acute illness or poor general health and refusal to participate.

**Fig 1:**
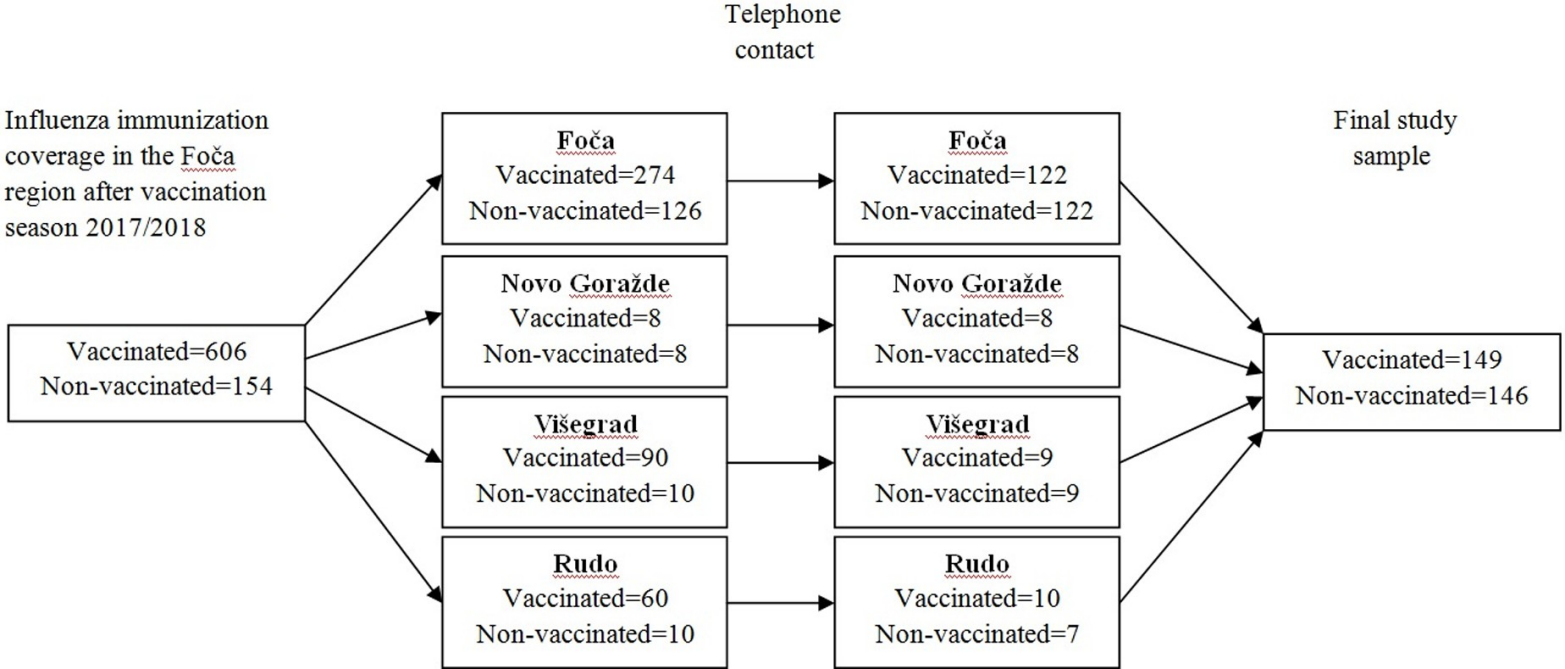
Selection of study participants.

Of 760 community-dwelling persons who had chronic diseases and were scheduled for influenza immunization in the Foča region. The selection of study participants was presented in Fig 1. After the completion of vaccination season 2017/2018 in the Foča region, 606 people with chronic diseases were vaccinated against influenza and 154 people remained non-vaccinated. These individuals were stratified according to town of residence. All 154 non-vaccinated people were contacted via telephone and invited to participate in this study. Of 154 people, we were able to reach 146 individuals. By using a computer-generated random selection, we identified a vaccinated person for each non-vaccinated individual in each town. After telephone contact, 149 people were included in this study. The total study sample included 295 people with chronic diseases.

The study was conducted from April to December 2018. All respondents provided written informed consent for participation. The Ethics Committee of the Faculty of Medicine, University of Belgrade approved the study (Approval no. 2650/VI-15).

### Instrument

The study instrument was the questionnaire Health Belief Model Applied to Influenza (HBMAI). The author of the HBMAI is Carolyn Blue [9]. However, at the time when our research team applied for permission to use the questionnaire, we learned that the author had passed. Therefore, it was not possible to receive permission to use the questionnaire from the author or her representative. Our research team was in contact with the authors who previously validated the HBMAI in Turkish language [10], from whom we obtained the original questionnaire in English. The obtained questionnaire had 1 item more than the 44-item HBMAI in the Turkish validation [10] (item #39 "I have the recommended dental exams in addition to visits for specific problems").

The survey was conducted through interviews with the participants at their homes. The HBMAI is composed of 45 items, which are divided in 7 different domains: 1-"Susceptibility" (comprising 7 items related to self-perceived sensitivity and risk of acquiring influenza); 2-"Seriousness" (covering 6 items related to the perception of potential consequences of being infected with the influenza virus); 3-"Benefits" (consisting of 6 items about the perception of advantages of being immunized against influenza); 4-"Barriers" (covering 8 items which reflect potential disadvantages of influenza immunization); 5-"Knowledge" (comprising 6 items which examine whether the respondents are familiar with some features of influenza); 6-"Health Motivation" (composed of 7 items related to general health-related behaviors); 7-"Cue to Action" (composed of 5 items which are related to the circumstance of being immunized against influenza due to recommendations) [6]. The truncated questions of the HBMAI are presented in S1 File.

Answers for all HBMAI items ranged from 1 to 5, where rank 1 indicated "strong disagreement" and rank 5 indicated "strong agreement". Items #2, #32 and #33 were scored in reverse, because they were formulated as negative statements. The summary score for all domains were calculated as a sum of ranks for all items from that domain. Higher scores indicated stronger agreement. The summary scores for the HBMAI domains were as follows: 7–35 points for "Susceptibility"; 6–30 points for "Seriousness"; 6–30 points for Benefits"; 8–40 points for "Barriers"; 6–30 points for "Knowledge"; 7–35 points for "Health Motivation"; 5–35 points for "Cue to Action". There is no summary score for the total HBMAI [6].

### Procedure

The original HBMAI questionnaire was developed in universal English language [9]. The translation of the English HBMAI was performed by two independent translators from the research team (*forward translation*). The two Serbian translations of the HBMAI were compared and discussed within the research team. Following this, one joined version of the questionnaire was created. Next, a third translator who was not involved in the forward translation, performed *back translation* from Serbian to English. After the back translation, this version of the questionnaire was compared to the original questionnaire. The research team concluded that the Serbian version of the HBMAI does not materially differ from the original questionnaire. Therefore, the Serbian and the English versions of the HBMAI were semantically and conceptually equivalent.

The Serbian version of the HBMAI was pilot tested on 10 adults, who confirmed that the questionnaire was clear and coherent, so no further modifications of the questionnaire were required.

### Data analysis

The data were analyzed using the JASP, version 0.14.0.0 (http://jasp-stat.org) and SPSS 20.0 statistical software package (SPSS Inc., Chicago, IL, USA).

First, we examined whether the structure of the HBMAI in Serbian mirrors the original HBMAI structure using the confirmatory factor analysis (CFA). The parameters which suggest acceptable fit on the CFA were as follows: goodness of fit index (GFI) >0.90, comparative fit index (CFI) >0.90, Tucker-Lewis index (TLI) >0.90, root mean square error of approximation (RMSEA) ≤ 0.08 and standardized root mean square residual (SRMR) ≤ 0.08 [12].

After acknowledging that the fit was not acceptable, we conducted the exploratory factor analysis (EFA). On EFA, the number of factors is observed to assess the existence of domains in a new population where the questionnaire is being administered. To make sure that we observed realistic number of domains in the dataset, the parallel analysis was conducted in addition to EFA [13, 14].

The internal consistency of the Serbian version of HBMAI was assessed using the coefficients Cronbach’s alpha and McDonald’s omega according to questionnaire domains [15]. The suggested adequate values of both coefficients are above 0.7 [15].

## Results and discussion

The study sample consisted of 295 persons. A total of 152 (51.5%) participants were men. The mean age of the participants was 63.3 ± 14.4 years. One half of participants (53.2%) were aged 65 and above. Most participants were retired (189, 64.1%), some were unemployed (58, 19.7%) and some were employed (47, 15.9%).

### Confirmatory factor analysis of the original structure

The results of the CFA are presented in Table 1. The CFA for the total questionnaire showed that none of the indices were acceptable (Table 1). The CFA according to domains of the original questionnaire suggested that the domain of "Knowledge" and "Susceptibility" had all indices except the GFI outside of the desirable range (Table 1). Because the indices were not appropriate, we have, therefore, conducted the EFA and subsequent parallel analysis.

**Table 1:** Parameters of the confirmatory factor analysis of the original Health Belief Model Applied to Influenza (HBMAI) among people with chronic diseases in Serbian language.

HBMAI Domains	Parameters on the confirmatory factor analysis				
	GFI	CFI	TLI	RMSEA	SRMR
Susceptibility	0.933	0.827	0.740	0.159	0.141
Seriousness	0.983	0.945	0.909	0.071	0.069
Benefits	0.992	0.995	0.991	0.039	0.056
Barriers	0.990	1.000	1.001	0.000	0.062
Knowledge	0.905	0.674	0.457	0.175	0.163
Health Motivation	0.982	0.983	0.975	0.044	0.065
Cue to Action	1.000	1.000	1.008	0.000	0.008
Total scale	0.640	0.684	0.662	0.093	0.124

GFI-goodness of fit index,CFI-comparative fit index, TLI-Tucker-Lewis index, RMSEA- root mean square error of approximation, SRMR-standardized root mean square residual

### Exploratory factor analysis

The parallel analysis suggested that the Serbian HBMAI has 6 domains, instead of the original 7 (S1 Table in S1 File). The results of the parallel analysis are shown in Table 2. The domains of "Barriers", "Health Motivation" and "Cue to Action" preserved their original structure. However, the domains of "Susceptibility", "Seriousness" and "Benefits" were partially modified i.e. several items were excluded. Namely, items #1 and #2 were excluded from the domain of "Susceptibility", while item #4 from this domain was moved to "Health Motivation". Items #11 and #13 were removed from the domain of "Seriousness", while item #8 from this domain was moved to the domain of "Susceptibility". Item #16 was excluded from the domain of "Benefits". Finally, the domain of "Knowledge" was completely omitted on the parallel analysis, and the only remaining item from this domain (#32) was moved to the domain of "Susceptibility".

**Table 2:** Exploratory factor analysis of the Health Belief Model Applied to Influenza in among people with chronic diseases in Serbian language (Q—question).

Domains	Factor loadings					
	Factor 1	Factor 2	Factor 3	Factor 4	Factor 5	Factor 6
Susceptibility						
Q 3	0.836					
Q 4					0.438	
Q 5	0.906					
Q 6	0.713					
Q 7	0.517					
Seriousness						
Q 8	0.644					
Q 9		0.478				
Q 10		0.577				
Q 11						
Q 12		0.610				
Benefits						
Q 14			0.760			
Q 15			0.694			
Q 17			0.696			
Q 18			0.758			
Q 19			0.494			
Barriers						
Q 20				0.604		
Q 21				0.748		
Q 22				0.812		
Q 23				0.886		
Q 24				0.790		
Q 25				0.638		
Q 26				0.776		
Q 27				0.578		
Knowledge						
Q 32	0.456					
Health Motivation						
Q 34					0.512	
Q 35					0.596	
Q 36					0.753	
Q 37					0.721	
Q 38					0.546	
Q 39					0.414	
Q 40					0.403	
Cue to Action						
Q 41						0.801
Q 42						0.801
Q 43						0.722
Q 44						0.492
Q 45						0.742

### Confirmatory factor analysis of the modified HBMAI

The new modified 6-factorial HBMAI was tested on the CFA. The results are shown in Table 3.

**Table 3:** Parameters of the confirmatory factor analysis of the modified Health Belief Model Applied to Influenza (mHBMAI) among people with chronic diseases in Serbian language.

Parameters	6-factorial mHBMAI according to EFA	6-factorial mHBMAI if item #4 deleted	6-factorial mHBMAI if items #4 and #8 deleted	6-factorial mHBMAI if items #4, #8 and #32 deleted
GFI	0.928	0.933	0.935	0.946
CFI	0.940	0.945	0.948	0.967
TLI	0.934	0.940	0.943	0.963
RMSEA	0.058	0.057	0.054	0.044
SRMR	0.088	0.087	0.085	0.078

EFA-exploratory factor analysis; GFI-goodness of fit index,CFI-comparative fit index, TLI-Tucker-Lewis index, RMSEA- root mean square error of approximation, SRMR-standardized root mean square residual

The indices suggested acceptable fit. However, we further inspected the modified domains, to examine whether items which were moved from one to the other domain logically fit within the construct. We observed that the CFA fit indices improve when items #4, #8 and #32 are removed sequentially from the new domains (Table 3). The model fit that was deemed the most appropriate on the CFA was observed when all three items (#4, #8 and #32) were removed. Based on fit indices, we accepted the final model with 6 domains and items #4, #8 and #32 removed. Factor loadings for the modified 6-factorial HBMAI without items #4, #8 and #32 are presented in Table 4. All factor loadings were about the suggested cut off of 0.4.

**Table 4:** Factor loadings and coefficient of determination for the modified Health Belief Model Applied to Influenza in Serbian language (Q—question).

Domains	Factor loading	95% Confidence interval	Standard error	Coefficient of determination R2	p
Susceptibility					
Q 3	0.811	0.721–0.900	0.046	0.569	0.001
Q 5	0.806	0.723–0.890	0.043	0.582	0.001
Q 6	0.951	0.904–0.998	0.050	0.703	0.001
Q 7	0.569	0.503–0.636	0.034	0.506	0.001
Seriousness					
Q 9	0.461	0.359–0.564	0.052	0.202	0.001
Q 10	0.636	0.521–0.752	0.059	0.424	0.001
Q 11	0.406	0.326–0.486	0.041	0.334	0.001
Q 12	0.531	0.436–0.625	0.048	0.505	0.001
Benefits					
Q 14	0.793	0.732–0.855	0.031	0.731	0.001
Q 15	0.683	0.624–0.743	0.031	0.538	0.001
Q 17	0.579	0.529–0.630	0.026	0.509	0.001
Q 18	0.823	0.759–0.887	0.033	0.692	0.001
Q 19	0.525	0.473–0.577	0.027	0.370	0.001
Barriers					
Q 20	0.572	0.527–0.617	0.023	0.530	0.001
Q 21	0.540	0.492–0.588	0.025	0.534	0.001
Q 22	0.531	0.480–0.582	0.026	0.532	0.001
Q 23	0.514	0.471–0.557	0.022	0.685	0.001
Q 24	0.500	0.449–0.550	0.026	0.478	0.001
Q 25	0.685	0.628–0.743	0.029	0.620	0.001
Q 26	0.535	0.483–0.588	0.027	0.484	0.001
Q 27	0.702	0.643–0.761	0.030	0.563	0.001
Health Motivation					
Q 34	0.430	0.368–0.491	0.031	0.415	0.001
Q 35	0.479	0.419–0.539	0.031	0.598	0.001
Q 36	0.533	0.462–0.604	0.036	0.520	0.001
Q 37	0.459	0.387–0.531	0.037	0.320	0.001
Q 38	0.452	0.388–0.516	0.033	0.417	0.001
Q 39	0.439	0.362–0.498	0.035	0.381	0.001
Q 40	0.539	0.473–0.615	0.039	0.415	0.001
Cue to Action					
Q 41	0.959	0.905–0.994	0.033	0.875	0.001
Q 42	0.923	0.868–0.988	0.033	0.831	0.001
Q 43	0.938	0.878–0.993	0.033	0.704	0.001
Q 44	0.884	0.832–0.934	0.025	0.697	0.001
Q 45	0.917	0.852–0.983	0.033	0.799	0.001

The CFA according to new domains of the Serbian HBMAI is shown in Table 5.

**Table 5:** Confirmatory factor analysis of the modified version of the Health Belief Model Applied to Influenza (mHBMAI) among people with chronic diseases in Serbian language.

Domains	Parameters on the confirmatory factor analysis				
	GFI	CFI	TLI	RMSEA	SRMR
m_Susceptibility	0.996	0.993	0.979	0.076	0.049
m_Seriousness	1.000	1.000	1.000	0.000	0.000
m_Benefits	0.995	0.996	0.993	0.041	0.051
Barriers	0.990	1.000	1.001	0.000	0.062
Health Motivation	0.982	0.983	0.975	0.044	0.065
Cue to action	1.000	1.000	1.008	0.000	0.008

m_x—modified domain; GFI-goodness of fit index,CFI-comparative fit index, TLI-Tucker-Lewis index, RMSEA- root mean square error of approximation, SRMR-standardized root mean square residual

### Internal consistency

The alpha and omega coefficients according to domains and questionnaire structure are showin in Table 6.

**Table 6:** Internal consistency of the modified Health Belief Model Applied to Influenza (HBMAI) in people with chronic diseases Serbian language.

Domains	Original HBMAI		Modified HBMAI	
	Cronbach’s α	McDonald’s ω	Cronbach’s α	McDonald’s ω
Susceptibility	0.606	0.746	0.849	0.860
Seriousness	0.674	0.693	0.632	0.647
Benefits	0.741	0.807	0.864	0.873
Barriers	0.912	0.909	0.912	0.909
Knowledge	0.519	0.493	/	/
Health Motivation	0.769	0.773	0.769	0.773
Cue to action	0.895	0.914	0.895	0.914

In the original structure, the alpha coefficients were appropriate for the domains of "Benefits", "Barriers", "Health Motivation" and "Cue to Action". Additionally, omega coeffcient was appropriate for "Susceptibility". In the new modified structure, alpha and omega coefficients were appropriate for all domains, except for "Seriousness". However, because the values of the coefficients for this domain were close to the suggested cut-offs, this domain was deemed acceptable.

## Discussion

This study is the first to examine the fit of a 7-domain HBMAI among people with chronic diseases. We observed that the original structure of the HBMAI did not fit the population who had chronic illnesses in the Foča region. We found that a more appropriate structure of the HBMAI in Serbian has 6 domains, instead of the original 7. The domain which was removed was the domain of "Knowledge" about influenza. We also observed that 3 out of 6 domains ("Susceptibility", "Seriousness" and "Benefits") were slightly modified i.e. not all the original items remained in the final accepted versions. The internal consistency of the domains in the proposed Serbian version of the HBMAI were acceptable.

To the best of our knowledge, the psychometric properties of the HBMAI beside English-speaking population, have been performed in Turkish [8] and Brazilian Portuguese languages [10]. For this reason, the comparison of our results may be limited. The modifications of the original structure of the HBMAI, similar to those observed in the Serbian version, have previously been reported [8, 10]. Specifically, the validations of the HBMAI conducted in the population of health care workers in Turkey [8] and general population in Brazil [10] suggested that the modified HBMAI was more appropriate for their respective populations. For example, the Brazilian Portuguese version of the HBMAI suggested that domains of "Seriousness", "Benefits" as well as "Knowledge" should be removed from the questionnaire [10]. In a similar manner, the Turkish version of the HBMAI suggested that both "Health Motivation" and "Knowledge" should be omitted [8]. While the Serbian version was the least restrictive in terms of domain removal compared to Turkish and Brazilian Portuguese versions, the only domain that was removed ("Knowledge") is consistent with the previous validations [8, 10]. It is interesting that in both general population (such as ours and in Brazil) and among health-care workers the domain of "Knowledge" about influenza was not acceptable component of the HBMAI. These findings suggest that the domain of "Knowledge" may not appropriate to measure the knowledge construct within the HBMAI. To adequately examine the knowledge about influenza, it is advised to entirely revise the knowledge domain of the HBMAI or develop an entirely separate and specific questionnaire which could be administered along with the HBMAI.

The examination of the construct validity of the Serbian HBMAI suggested that domains of "Susceptibility", "Seriousness" and "Benefits" should not entirely keep the original items. Two items were completely removed from "Susceptibility" (#1 "Working with multiple people each day increases my chances of getting the flu" and #2 "Only people over 65 years of age get the flu"). The omission of item #1 can be explained by the composition of the study population. Specifically, approximately 83% of people in our sample were not employed (i.e. they were retired or unemployed) and do not have a habitual activity of working with multiple people. This could also explain the omission of item #11 "Having the flu would make daily activities more difficult" from the "Seriousness" domain and item #16 "Getting a flu shot will prevent me from being absent from work" from the "Benefits" domain. On the other hand, the item #2 differs from the other items in the "Susceptibility" domain. Namely, all items refer to the individual perception of flu susceptibility, while item #2 refers to a general statement which is probably not perceived personally. Finally, item #13 "Flu can be a serious disease" was removed from the "Seriousness" domain. This item is similarly worded to item #12 which was retained ("If I get the flu, it would be more serious than other diseases") and it is possible that both items were perceived in a parallel manner and item #13 was, therefore, redundant.

The examination of the internal consistency of the 6 domains of HBMAI in Serbian as measured by Cronbach’s alpha and McDonald’s omega coefficients suggested that 5 domains had desirable metrics, while one domain ("Seriousness") had slightly lower values. The HBMAI validation in Turkish language showed that all domains had very high alpha coefficients [10]. Previous studies did not assess internal consistency using the omega coefficient [8, 10], which has been increasingly relevant in the psychometric assessment because it is deemed more reliable than the alpha coefficient [16].

This study has some limitations. While we have included people throughout the Foča region, the sample size in this study was relatively small. Some authors suggest that the ratio between the questionnaire items and number of study participants should be at least 5 [17]. In this study, the item-to-participant ratio was 1:6.5 (45 items: 295 participants), which could be acceptable to reduce bias in conducting the EFA. In addition, we performed the parallel analysis to remedy the issue of small sample size to obtain the realistic number of factors. While we have performed face validity and construct validity, we did not compare the HBMAI to other similar questionnaires nor with potential clinical parameters of the study participants. This means, that the concurrent and criterion validity are missing. Finally, this study is limited by the absence of test-retest assessment. Thus, we were not able to examine the reproducibility of the HBMAI in Serbian at two time points.

Despite these limitations, our findings highlight that the existing questionnaires need to be validated and their psychometric properties need to be assessed in various population groups. This is important, because collection of relevant data in clinical practice should be systematically organized in order to obtain accurate data. Having accurate data in clinical practice is essential to reorganize, adjust and adapt health care delivery and health promotion strategies. People who have chronic diseases are at risk of developing health complications due to influenza. Their beliefs about influenza vaccination are strongly related to compliance with vaccination. Health care workers need to continuously promote and support influenza immunization, especially in circumstances of misconceptions about vaccines caused by the rapid spread of information of problematic quality. To achieve this, accurate assessment of people beliefs about influenza should be prioritized.

## Conclusions

In conclusion, this study suggested that the HBMAI in Serbian language, tested on people who have chronic diseases, has 32 items and 6 domains (Susceptibility, Seriousness, Benefits, Barriers, Health Motivation and Cue to Action). The domain of Knowledge about influenza was not psychometrically suitable for this construct and should be either revised or redeveloped. The domains of the modified HBMAI have acceptable internal consistency. This modified version of the HBMAI is recommended for use in research about influenza among people with chronic diseases in Serbian language.

## Supporting information

S1 Dataset (XLSX)

S1 File (DOC)
