# Supplementary material for: Validity of the Health Belief Model Applied to Influenza among people with chronic diseases: Is it time to develop a new knowledge domain?
Source: PLoS One. 2022 Sep 15;17(9):e0274739. doi: 10.1371/journal.pone.0274739 (PMC9477327; doi:10.1371/journal.pone.0274739)
Supplement: S1 File — (DOC) [file pone.0274739.s002.doc]

Validity of the health belief model applied to influenza in persons with chronic diseases: is it time to develop a new knowledge domain?

Supporting information

**S1 Table**. **Comparison of eigenvalues on the exploratory factor analysis and parallel analysis of the Health Belief Model Applied to Influenza in Serbian language among people with chronic diseases.** HBMAI - Health Belief Model Applied to Influenza; Factors are true if the eigenvalues on the parallel analysis are lower than the eigenvalues on the exploratory factor analysis

**S1 File. Health Belief Model Applied to Influenza questionnaire.**

**S1 Table**. **Comparison of eigenvalues on the exploratory factor analysis and parallel analysis of the Health Belief Model Applied to Influenza in Serbian language among people with chronic diseases.** HBMAI - Health Belief Model Applied to Influenza; Factors are true if the eigenvalues on the parallel analysis are lower than the eigenvalues on the exploratory factor analysis

| Factors of the HBMAI | Eigenvalues | |
| --- | --- | --- |
|  | Exploratory factor analysis | Parallel analysis |
| 1 | 9.752 | 1.915 |
| 2 | 5.051 | 1.803 |
| 3 | 3.413 | 1.735 |
| 4 | 2.766 | 1.677 |
| 5 | 2.226 | 1.611 |
| 6 | 1.932 | 1.558 |
| 7 | 1.487 | 1.507 |

Legend: HBMAI - Health Belief Model Applied to Influenza; Factors are true if the eigenvalues on the parallel analysis are lower than the eigenvalues on the exploratory factor analysis

**S1 File. Health Belief Model Applied to Influenza questionnaire.**

Items are truncated. Full items are available from the publisher.

1 = Strongly disagree (SD)

2 = Disagree (D)

3 = Neither agree or disagree (N)

4 = Agree (A)

5 = Strongly agree (SA)

S*usceptibility* **(Circle one number)**

**SD D N A SA**

1. Working with multiple people... 1 2 3 4 5

2. Only people over 65 years... 1 2 3 4 5

3. My chances of getting the flu... 1 2 3 4 5

4. Healthy people can... 1 2 3 4 5

5. I feel the chances of getting the flu... 1 2 3 4 5

6. I worry a lot about... 1 2 3 4 5

7. I will get the flu... 1 2 3 4 5

*Seriousness*

8. The thought of getting... 1 2 3 4 5

9. If I get the flu, my job would... 1 2 3 4 5

10. Getting the flu would... 1 2 3 4 5

11. Having the flu would make daily activities... 1 2 3 4 5

12. If I got the flu, it would be... 1 2 3 4 5

13. Flu can be a... 1 2 3 4 5

*Benefits*

14. Getting a flu shot will prevent me... 1 2 3 4 5

15. Getting a flu shot will protect others... 1 2 3 4 5

16. Getting a flu shot will prevent my... 1 2 3 4 5

17. I have a lot to gain by... 1 2 3 4 5

18. I would not be afraid... 1 2 3 4 5

19. Having a chronic illness... 1 2 3 4 5

*Barriers*

20. Getting a flu shot is not... 1 2 3 4 5

21. In order to get a flu shot, I would... 1 2 3 4 5

22. Getting a flu shot can... 1 2 3 4 5

23. Getting a flu shot is... 1 2 3 4 5

24. Getting a flu shot interferes with... 1 2 3 4 5

25. There are too many risks... 1 2 3 4 5

26. It costs too much.... 1 2 3 4 5

27. I am concerned about having... 1 2 3 4 5

*Knowledge*

28. People get the flu from eating... 1 2 3 4 5

29. People get the flu from breathing... 1 2 3 4 5

30. The flu lasts... 1 2 3 4 5

31. Getting the flu can cause more... 1 2 3 4 5

32. One can get the flu from... 1 2 3 4 5

33. People often get sick from... 1 2 3 4 5

*Health Motivation*

34. I eat a... 1 2 3 4 5

35. I follow medical orders because.... 1 2 3 4 5

36. I frequently do things on my own... 1 2 3 4 5

37. I search for new information... 1 2 3 4 5

38. I have the recommended yearly physical... 1 2 3 4 5

39. I have the recommended dental exams... 1 2 3 4 5

40. I exercise regularly... 1 2 3 4 5

*Cue to Action*

41. I decided to get a flu vaccine when... 1 2 3 4 5

42. I got the flu vaccine because a friend... 1 2 3 4 5

43. I got the flu vaccine because my doctor... 1 2 3 4 5

44. I got the flu vaccine because my supervisor... 1 2 3 4 5

45. I got the flu vaccine after hearing an... 1 2 3 4 5
